# Upper gastrointestinal bleeding related to an eroded gastrosplenic collateral lately after splenic artery embolization

**DOI:** 10.1186/s42155-022-00322-1

**Published:** 2022-08-19

**Authors:** Anna Van Thillo, Pieter-Jan Buyck, Stijn Van Gool, Cléo Croonen, Geert Maleux

**Affiliations:** 1 Department of Gastroenterology, Algemeen Ziekenhuis Turnhout, Turnhout, Belgium; 2 Department of Gastroenterology and Hepatology, University Hospitals KU Leuven, Leuven, Belgium; 3 Department of Radiology, University Hospitals KU Leuven, Leuven, Belgium; 4 University Hospitals KU Leuven, Leuven, Belgium

**Keywords:** Upper gastrointestinal bleeding, Embolization, Splenic artery, Trauma

## Abstract

**Background:**

Upper gastrointestinal, non-variceal haemorrhage can be related to various etiologies, including peptic ulcer, neoplasm, gastritis, Dieulafoy lesions and other, rare underlying diseases. Here, we describe another, yet unreported etiology of gastric bleeding.

**Case presentation:**

A 49-year-old man presented with melena; gastroscopy revealed blood in the stomach without active bleeding source. Computed tomography angiography demonstrated a cluster of enlarged gastrosplenic arterial collaterals in the gastric wall and coils in the splenic artery, related to an embolization procedure 30-years ago for splenic trauma. Definitive treatment included catheter-directed glue embolization of the left gastric artery and the enlarged gastrosplenic collaterals. The postinterventional course was uneventful and no recurrence of upper gastrointestinal bleeding was noted after 6 months of follow-up.

**Conclusions:**

Upper gastrointestinal bleeding associated with eroded gastrosplenic collaterals, related to previous splenic artery embolization, can be successfully treated with glue-embolization.

## Introduction

Non-variceal, upper gastrointestinal, haemorrhage can be related to various etiologies, including peptic ulcer, neoplasm, gastritis, Dieulafoy lesions and other, rare underlying diseases (Augustin et al., 2019). In this report, we describe another, yet unreported etiology of non-variceal upper gastrointestinal bleeding in a patient who previously underwent selective splenic artery embolization related to a traumatic event.

## Case report

**Fig. 1:**
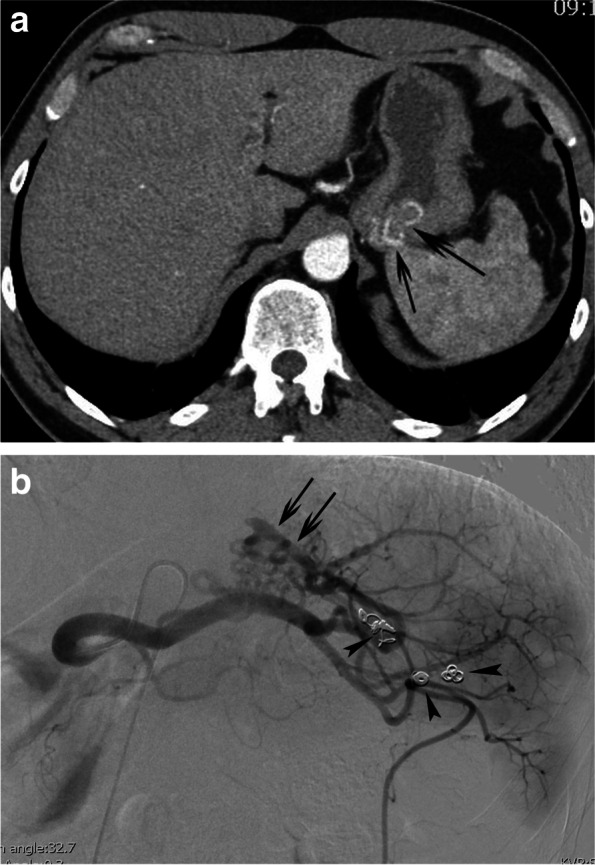
**a** Arterial-phase, contrast-enhanced CT reveals a cluster of enlarged arteries (black arrows) in the posterior portion of the gastric fundus. **b** Corresponding selective splenic artery angiography shows a cluster of hypertrophied arterial collaterals (black arrows), between the splenic artery main branch and the upper splenic pole segmental arteries

**Fig. 2:**
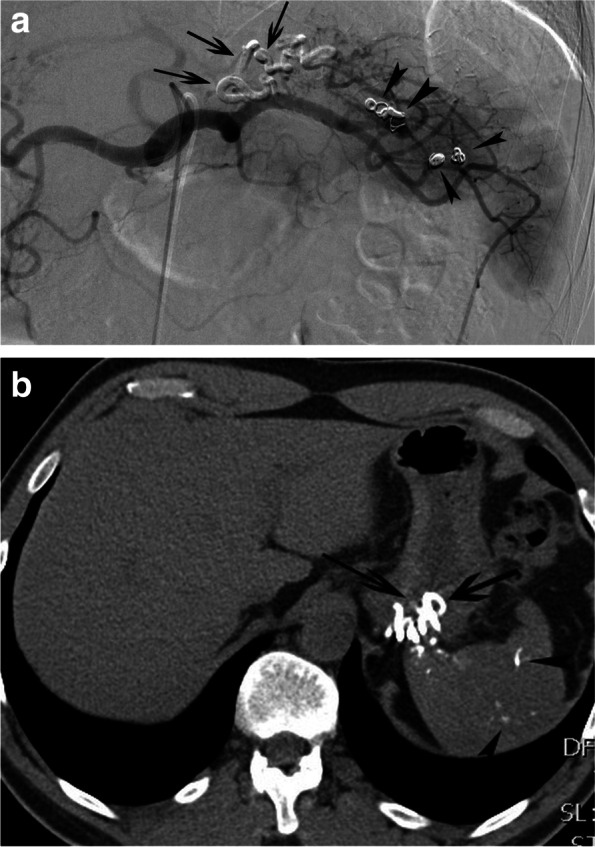
**a** Selective splenic artery angiography after glue-embolization shows the cast of the glue, completely occluding the cluster of hypertrophied arterial collaterals in the posterior gastric wall. **b** Follow-up unenhanced CT reveals the dense cast of glue in the cluster of hypertrophied arterial collaterals (black arrows). Note also some droplets of glue (arrowheads), migrated into splenic parenchyma

**Fig. 3:**
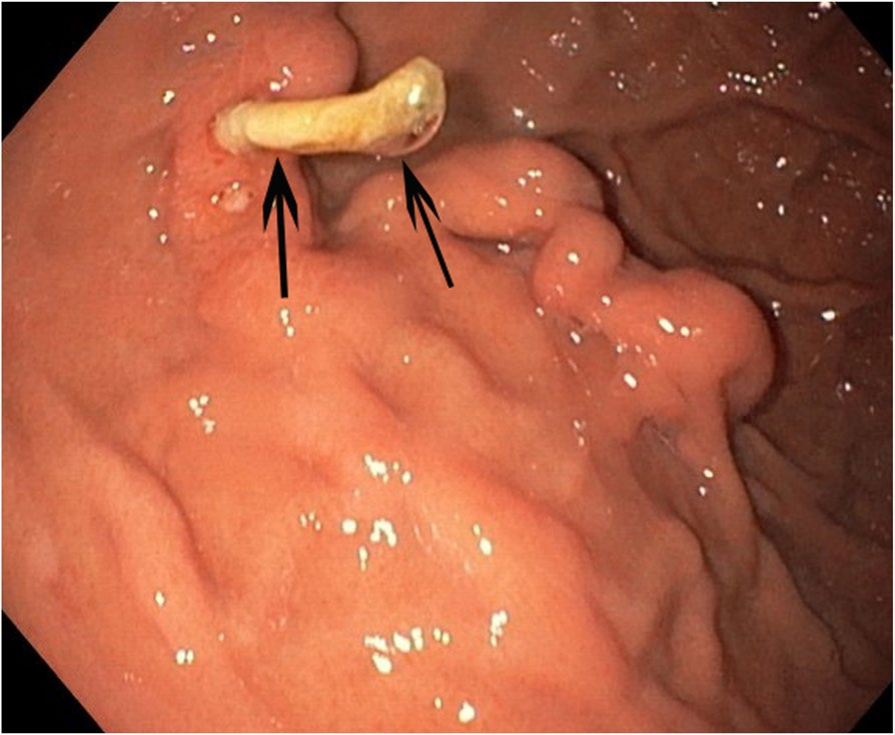
Follow-up gastroscopy one month after glue-embolization shows part of the glue-cast (arrows) protruding into the gastric lumen. No other mucosal lesions could be identified

A 49-year-old military male presented with melena since 5 days; furthermore other symptoms of anemia, including fatigue, dizziness and palpitations on exertion were noted. There was no history of previous gastro-intestinal bleeding and there was only sporadic use of alcohol. Clinical assessment revealed a stable patient with low blood pressure (107/67 mmHg) and normal pulse frequency (67 pulses/min). Laboratory analysis showed macrocytic anemia (haemoglobin 6.2 g/dl); other laboratory findings were normal, including coagulation, ionogram, liver and renal function. Upper endoscopy revealed a large blood clot in the fundus without active bleeding and a gastric fundal varix with an erosion was suspected. The patient was treated initially with administration of 2 units of packed cells, terlipressin (6 mg/24 hours) and a proton pump inhibitor. Contrast-enhanced triple-phase computed tomography (CT) was performed, showing a normal aspect of the liver without signs of portal hypertension and a cluster of enlarged arteries in the gastric wall (Fig. 1a) without clear contrastextravasation. In addition, several metallic coils were found in and around the splenic hilum. No ectopic splenic tissue could be visualized in or around the stomach. At that time, the patient remembered to be treated by catheter-directed embolization for splenic trauma 30 years ago. It was hypothesized that gastro-splenic collaterals developed over the years related to segmental splenic artery coil-embolization. Subsequently, the patient was referred to interventional radiology for further and definitive management. Under local anesthesia, through a 4 French sheath, a Simmons 1 catheter (Glidecatheter, Terumo Europe, Leuven, Belgium) was navigated into the celiac trunk. Contrast injection revealed enlarged gastrosplenic arterial collaterals between the main splenic artery and the upper pole splenic endbranches, bypassing the coil-occluded distal segment of the main splenic artery (Fig. 1b). These gastrosplenic collaterals were superselectively cannulated with use of a microcatheter (Progreat 2.4, Terumo Europe, Leuven, Belgium) and embolized with a 1/3 mixture of enbycrylate (Glubran, GEM srl, Viareggio, Italy) and Lipiodol (Guerbet, Villepinte, France). Completion angiography showed complete occlusion of the gastrosplenic collaterals and some droplets of glue migrating into the splenic upper pole (Fig. 2a). Computed tomography 2 days later, revealed a cast of glue in the hypertrophied collaterals in the gastric wall and some droplets of glue in the splenic parenchyma (Fig. 2b). The postinterventional course was uneventful without clinical evidence for gastric or splenic ischemia; no episode of bleeding recurrence was noted. A follow-up upper endoscopy 6 weeks after the embolization showed a persistent tangle of thick folds and a strand coming out of the erosion: most probably a part of the embolization cast (Fig. 3). No recurrence of gastrointestinal bleeding was noted after 6 months of clinical follow-up.

## Discussion

This case report demonstrates a, yet unreported, very late adverse event of splenic artery coil-embolization for splenic trauma. Gastrosplenic collaterals developed in the gastric wall over 30 years and eroded into the gastric lumen with subsequent upper gastrointestinal bleeding. However, it is unclear if this late bleeding complication after segmental splenic artery coil embolization may also occur after main splenic artery occlusion, as nowadays most often performed to manage splenic trauma (Quencer & Smith, 2019). These submucosal collaterals may be misinterpreted as varices on upper endoscopy (Mnatzakanian et al., 2008) and endoscopic injection of sclerosant agents might be a suboptimal treatment with potential non-target migration of the injected material. Enlarged gastrosplenic collaterals have been described in patients with congenital absence of the splenic artery (Spriggs, 1984) and in patients with main splenic artery occlusion related to different etiologies, including blunt abdominal trauma (Baron et al., 2000), splenic artery surgery, including aneurysmectomy and main splenic artery ligation after liver transplantation (Worthley et al., 2003; Keramidas et al., 1984). Irrespective of the underlying etiology of absence or occlusion of the main splenic artery, gastrosplenic collaterals may develop in the gastric wall and erode in the gastric lumen, resulting in severe intestinal bleeding. Abdominal CT study revealed these hypertrophied collaterals in the gastric wall and prompted referral to interventional radiology for embolization.

Embolization was performed with glue, occluding the whole cluster of gastric wall collaterals, as confirmed by follow-up CT and upper endoscopy, showing a part of the cast protruding through the gastric mucosa into the gastric lumen. The strand of glue cast, clearly visible on follow-up endoscopy, most probably completely occluded the index bleeding point. In addition, despite a substantial amount of glue injected and gastrosplenic collaterals embolized, no clinical, radiologic or endoscopic signs of gastric ischemia could be identified.

## Conclusion

Gastrosplenic collaterals may be formed in the gastric submucosa many years after a successful proximal splenic artery coil-embolization for the management of splenic trauma and may erode in the gastric lumen resulting in severe upper gastrointestinal bleeding. Subsequently, patients, coil-embolized for splenic trauma should be consented for the potential of late upper gastrointestinal bleeding. Submucosal gastrosplenic collaterals may be misinterpreted as gastric varices on endoscopy; catheter-directed glue embolization seems to be safe and effective to definitively stop this type of upper gastrointestinal bleeding.

## Data Availability

The datasets used and/or analysed during the current study are available from the corresponding author on reasonable request.

## Abbreviation

CT: Computed tomography
